# Identification of a Novel Mutation in a Family with Pseudohypoparathyroidism Type 1a

**DOI:** 10.1155/2018/7813591

**Published:** 2018-01-22

**Authors:** Adelaide Moutinho, Rosa Carvalho, Rita Ferreira Reis, Sandra Tavares

**Affiliations:** ^1^Department of Internal Medicine, Hospital de Chaves, Centro Hospitalar de Trás-os-Montes e Alto Douro, Chaves, Portugal; ^2^Department of Internal Medicine, Hospital de Braga, Braga, Portugal; ^3^Department of Internal Medicine, Hospital de Lamego, Centro Hospitalar de Trás-os-Montes e Alto Douro, Lamego, Portugal

## Abstract

**Introduction:**

Pseudohypoparathyroidism type 1a is caused by GNAS mutations leading to target organ resistance to multiple hormones rather than parathyroid hormone, resulting not only in hypocalcemia, but also in Albright's hereditary osteodystrophy phenotype.

**Materials and Methods:**

DNA sequencing of the GNAS gene identified a novel heterozygous mutation in peripheral blood leukocytes in the family presented in this case report.

**Results:**

We present a case of a 25-year-old woman with pseudohypoparathyroidism type 1a admitted with seizures, whose family presents an autosomal dominant transmission of a novel heterozygous GNAS mutation (c.524_530+3del).

**Conclusion:**

Pseudohypoparathyroidism type 1a is mostly caused by inactivating GNAS mutations that have been gradually reported in the literature that lead to a typical and complex clinical phenotype and resistance to multiple hormones. The deletion caused by the mutation identified in the presented case has not been reported previously.

## 1. Introduction

Pseudohypoparathyroidism (PHP) is a rare disorder characterized by target organ resistance to parathyroid hormone (PTH), resulting in hypocalcemia and hyperphosphatemia [1, 2]. PHP type 1a is due to a heterozygous loss of function of the alpha subunit of a G protein (Gs*α*), due to a GNAS mutation on the maternal allele of the chromosome 20q13.3, with autosomal dominant inheritance [3, 4]. This intracellular protein is responsible for the production of cyclic AMP (cAMP) in response to PTH, and the reduced G protein activity is the molecular basis for hormone resistance in this disorder [3]. PHP type 1a is characterized by the expression of the Gs*α* isoform only of the paternal GNAS gene, with resistance to other hormones rather than PTH and by the phenotype of Albright's hereditary osteodystrophy (AHO) with round facies, short stature, obesity, subcutaneous ossifications, brachydactyly, and in some cases mental retardation [3–5].

## 2. Case Presentation

A 25-year-old woman was admitted to the Emergency Service after a generalized tonic-clonic seizure. She had a history of hypoparathyroidism, hypothyroidism, and mild retardation, being medicated with calcium carbonate 9000 mg plus cholecalciferol 2400 U/day, levothyroxine 0,1 mg/day, and desogestrel/ethinylestradiol 0,15/0,02 mg/day. She had multiple hospital admissions with seizures because of lack of therapeutic compliance and had repeatedly refused further investigation of the underlying disease or regular outpatient follow-up.

**Figure 1:**
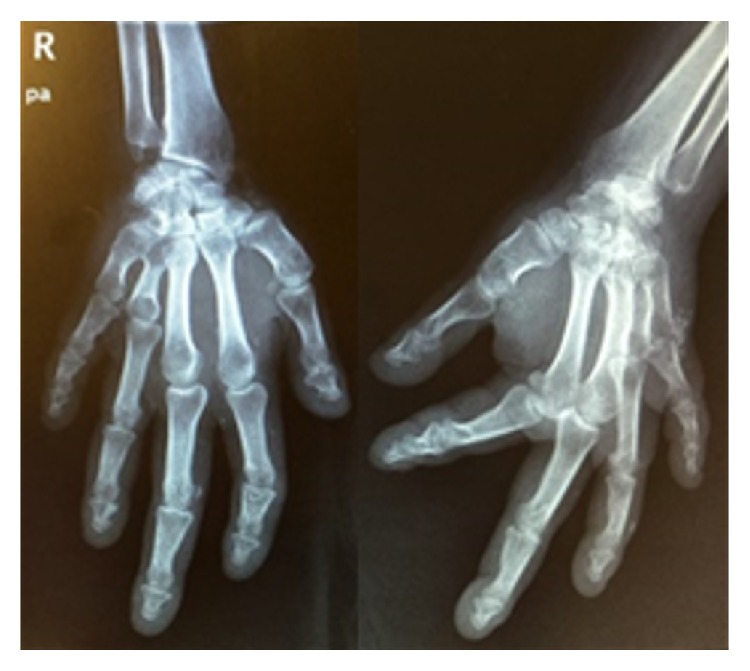
Radiography of both hands, revealing brachydactyly of the fourth and fifth metacarpals.

Physical examination showed a short stature, obesity (BMI 35 kg/m^2^), dental hypoplasia, round facies, and brachydactyly of the fourth and fifth metacarpals (Figure 1) and metatarsals. At admission she was conscious, with Glasgow Coma Scale of 15 points, had normal vitals (blood pressure 120/60 mmHg; heart rate 68 beats per minute; respiratory rate 16 cycles per minute; O_2_ saturation of 96%; FiO_2_ 21%), with no fever (temperature 36,7°C). She had positive Chovstek and Trousseau signs, without tetany. Her pulmonary and cardiac auscultations were normal. The neurologic examination revealed no focal signs, no photophobia, and negative Kernig's and Brudzinski's signs.

**Figure 2:**
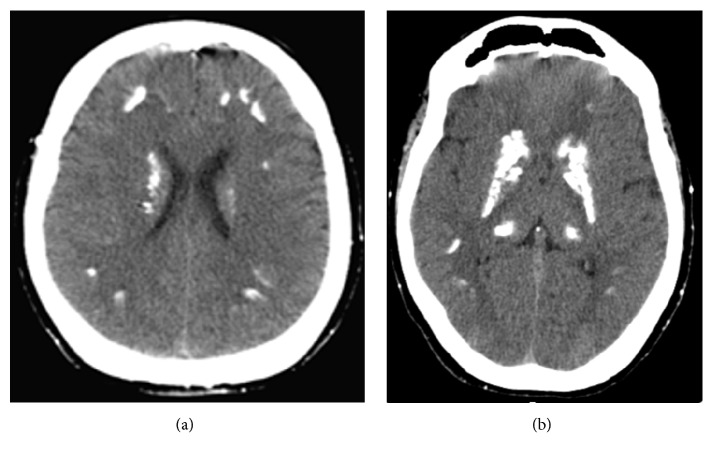
Axial cuts of CT scan, revealing diffuse subcortical frontoparietal (a) and striatum capsularis and thalamus calcifications (b).

Laboratory tests revealed severe hypocalcemia (serum calcium with albumin correction 5,5 mg/dL; normal range (NR) 8,6–10,0 mg/dL), hyperphosphatemia (6,7 mg/dL; NR 2,7–4,5 mg/dL), and high PTH (160,1 pg/mL; NR 10–65 pg/mL), with normal vitamin D (37 ng/mL; NR 30–100 ng/mL) and low calcium in the 24-hour urine collection (23,9 mg/24 h; NR 100–300 mg/24 h). The thyroid-stimulating hormone (TSH) was normal (4,04 mIU/L; NR 0,27–4,2 mIU/L), with free thyroxine of 16,53 pmol/L (NR 1,17–21,7 pmol/L). The CT scan revealed diffuse subcortical frontoparietotemporal, striatum capsularis, and thalamus calcifications (Figure 2), without other changes.

Her family history revealed an aunt and a sister with clinical diagnosis hypoparathyroidism and AHO, as well as two nephews, without further investigation, and the presence of AHO in her mother, without further investigation; her older brother had a fatal respiratory infection in infancy. Our subject's one-year-old son had AHO and hypoparathyroidism as well. Considering this presentation, we assumed that all individuals should have pseudohypoparathyroidism as well.

**Figure 3:**
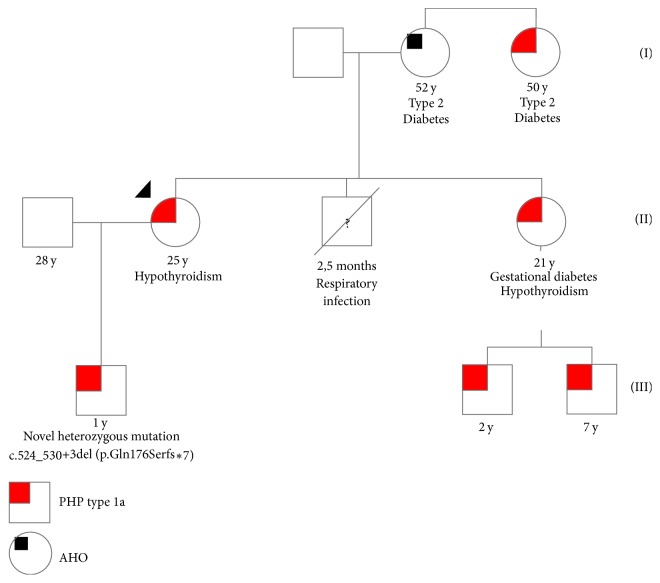
Genogram of the subject's family. Our subject is identified by the black arrow-head. y: year(s) old; PHP: pseudohypoparathyroidism; AHO: Albright's hereditary osteodystrophy.

Based on the clinical features of AHO plus the pseudohypoparathyroidism with hypocalcemia, hyperphosphatemia, and tissue resistance to the increased level of PTH, she was diagnosed with PHP type 1a. Facing her family history and to support our diagnosis, and after explaining to the patient the implications of the diagnosis for both her and her child, we were able to obtain consent to further investigation that she had refused before. Therefore, we performed a genetic study with DNA analysis of the GNAS gene extracted from peripheral blood leukocytes with identification of the novel mutation c.524_530+3del (p.Gln176Serfs*∗*7) in heterozygosity, in the location 20q13.32-exon 6, both in our patient and in her son. The genogram (Figure 3) suggests an autosomal dominant inheritance with maternal derived transmission. We were not able to perform DNA sequencing of the GNAS gene of any other family member.

## 3. Discussion

PHP type 1a is caused mostly by maternal heterozygous inactivating mutations in the GNAS gene leading to a complex clinical phenotype that includes resistance to multiple hormones, intellectual disability, and AHO [1, 4]. This phenotype is most likely explained by the fact that some tissues (thyroid, pituitary, renal proximal tubules, and gonads) express Gs*α* predominantly from the maternal allele, while the paternal is silenced through yet unknown mechanisms. In PHP type 1a, inactivating mutations on the maternal allele will result in little or no production of this subunit with haploinsufficiency of Gs*α*, resulting in multiple hormone resistance within these specific tissues [3]. The resistance to PTH leads to hypocalcemia and hyperphosphatemia, and as calcium plays an essential role in stabilizing the cell membrane, there is an increased risk of seizures, as happened with our patient [2].

Lemos and Thakker [6] identified 343 kindreds with heterozygous Gs-alpha germline mutations in the literature that yielded a total of 176 different germline mutations, without unknown prevalence, but with some acknowledged mutational hot spots, as exon 1 and codons 189-190 in exon 7. A novel mutation was identified in both our patient and her son in exon 6. The genogram suggests the autosomal dominant maternal transmission to both our patient and her sister. However, we were not able to test our patient's relatives which was a limitation in this case.

In conclusion, there are over 340 reported GNAS mutations leading to PHP type 1a [1], and the identification of the causative mutation in the index case may be useful for screening other family members avoiding late diagnosis or misdiagnosis and for prenatal counseling both to our patient and to her sister and in the future for our patient's son and nephews.
